# Projecting the Potential Budget Impact Analysis of Paliperidone Palmitate in Egyptian Adult Patients with Schizophrenia

**DOI:** 10.36469/001c.83240

**Published:** 2023-08-17

**Authors:** Gihan Elsisi, Mohamed Ezzat, Mohamed Ramadan

**Affiliations:** 1 HTA Office, LLC, Cairo, Egypt; 2 Economics Department American University in Cairo, Cairo, Egypt; 3 Health Insurance Organization, Cairo, Egypt; 4 Faculty of Medicine Alazhar University, Cairo, Egypt

**Keywords:** budget impact analysis, paliperidone palmitate, Egypt, schizophrenia, societal perspective

## Abstract

**Background:** Schizophrenia is a serious mental disorder that has greater negative consequences on role functioning than many other severe chronic diseases.

**Objective:** We evaluated the economic impact of long-acting injections of paliperidone palmitate (PP) vs daily oral antipsychotics to treat chronic schizophrenia from a societal perspective over a 2-year period.

**Methods:** A static budget impact model was developed to compare PP with daily oral antipsychotics (risperidone, olanzapine, and aripiprazole) in the treatment of patients with chronic schizophrenia. Our study included treatments used during relapse and hospitalization, validated by an expert panel. The clinical parameters were extracted from the PRIDE trial. Direct medical costs and indirect costs were measured. The unit cost of drug acquisition for all medications was extracted from the public sector. One-way sensitivity analyses were conducted.

**Results:** The target population in our model was estimated to be 142 incident patients. In the first year, the total drug costs in Egyptian pounds (EGP) for PP and oral antipsychotics were £2.7 million and £724 004, respectively, while the total medical costs for PP and oral antipsychotics were £3 million and £5.6 million, respectively. In the second year, the total drug costs for PP and oral antipsychotics were £2.7 million and £724 004, respectively, while the total medical costs for PP and oral antipsychotics were £3 million and £5 million, respectively. The total costs for PP (£11.6 million) over 2 years were less than those of oral antipsychotics without PP (£12.7 million). PP produced an estimated budget savings of £1 046 561 (budget savings per patient per year, £3667). In addition, PP resulted in the avoidance of 18 hospitalizations per year compared with the without-PP arm. Sensitivity analyses showed that the percent of hospitalizations for both oral antipsychotics and PP had the greatest impact on the results.

**Conclusion:** The lower hospitalization rates associated with PP offset the increase in drug costs. PP may potentially be cost-saving compared with the standard of care in chronic schizophrenia in Egyptian representative healthcare settings. Policy makers may consider this approach to improve patient outcomes and budget sustainability.

## BACKGROUND

Mental disorders, including depressive disorders, bipolar disorder, schizophrenia, anxiety disorders, and autism spectrum disorder, are major causes of disability-adjusted life-years (DALYs), affecting more than 1 billion people worldwide.^1^ Furthermore, they account for 7% of the global burden of disease, as measured by DALYs, and 19% of all years lived with disability.^1^ The incidence rates of mental disorders are increasing due to an aging population and the deterioration in infrastructure and public health services. The world suffers from an increasing burden of mental disorders and a widening treatment gap: approximately 450 million people suffer from a mental disorder,^2^ and only a small minority receive treatment. The prevalence of mental disorders has been estimated to be 16.93% in the Egyptian adult population.^2^ All mental disorders of clinical concern are reimbursed in the Egyptian health insurance system.^2^

Schizophrenia is a serious mental disorder that has greater negative consequences on role functioning than many other severe chronic diseases.^3^ It is a disabling mental illness that presents with mixed symptoms.^4^ It may result in a combination of hallucinations, delusions, and extremely disordered thinking and behavior that impairs daily functioning. Many patients experience an erratic journey that often involves several psychotic relapses, frequently resulting in hospital admission or incarceration.^5^ Schizophrenia is also characterized by periods of largely partial remission alternating with periods of relapse in approximately 75% of all patients. The estimated relapse rate over 7 to 12 months following clinical stabilization in patients continuing antipsychotic medications is 27%. Approximately 80% of patients relapse within 5 years of the initial event, with no more than 20% of patients recovering completely after the first event.^6,7^ Patients with schizophrenia are 2 to 3 times more likely to die early than the general population.^8^

People with schizophrenia require lifelong treatment. The disease exerts a tremendous negative impact on health care in terms of cost, service provision, and support systems.^9^ The average prevalence of psychiatric work loss days is 6 days per month per 100 workers, and the incidence of work cutback days is 31 days per month per 100 workers.^3^ Schizophrenia is the most common mental disorder within inpatient facilities and mental hospitals in Egypt. The length of stay for patients admitted to mental hospitals is longer than for those in community-based units.^2^ Patients’ quality of life is significantly affected by schizophrenia symptoms, which indicate patient dissatisfaction and poorer quality of life.^10^ The World Health Organization Assessment Instrument for Mental Health Systems (WHO-AIMS) has been used to collect data on mental health problems in Egypt, thus enabling the development of information-based mental health plans with clear baseline information and targets.^2^ It has been found that 2% of governmental health spending in Egypt was directed toward mental health. There are 62 outpatient mental health facilities available in Egypt. In 2004, these facilities treated 254 individuals per 100 000 citizens. Females, some of whom are the breadwinners in their household, are more commonly found in outpatient facilities and mental hospitals than males. Furthermore, there is no patient association for mental disorders in Egypt.^2^ All these findings highlight the large burden of schizophrenia in Egypt and the need for appropriate treatments.

It is often difficult to achieve success with pharmacological treatment mainly due to poor adherence, which has been observed in an average of 41% of schizophrenia cases.^11^ It is important to find approaches to improve both adherence and response rates. Long-acting injections (LAIs) of antipsychotics are the most widely used approach to combat nonadherence in common clinical practice, although their use is generally limited to patients who are partially or fully noncompliant with oral medications.^12^ The Scottish Medicines Consortium has advised National Health Service (NHS) Boards and Area Drug and Therapeutic Committees on paliperidone palmitate (PP) at doses of 50 mg, 75 mg, 100 mg, and 150 mg in the form of prolonged release suspensions for injection use to treat schizophrenia in NHS Scotland.^1^ One meta-analysis demonstrated the superior efficacy of long-acting vs oral antipsychotics in reducing relapse, and a subgroup analysis in another meta-analysis indicated that long-acting first-generation antipsychotics were more effective than oral medications.^13,14^

The Paliperidone Palmitate Research in Demonstrating Effectiveness (PRIDE) study compared the effectiveness of once-monthly PP with daily oral antipsychotics on treatment failure in 450 adults with schizophrenia and a history of incarceration. This was a 15-month, randomized, multicenter study. Time to first treatment failure was determined by a blinded event-monitoring board. They found that in real-world management of schizophrenia, PP was associated with a significant delay in time to first treatment failure compared with oral antipsychotics due to the reduced arrest/ incarceration and psychiatric hospitalization rates associated with PP.^15^

Currently, the economic impact of LAIs of PP to treat chronic schizophrenia in real-world practice has not yet been determined in Egypt. Thus, our main objective is to compare the economic impact of LAIs of PP in chronic schizophrenia with that of daily oral antipsychotics from a societal perspective over a period of 2 years to guide policy makers and decision makers to provide the best available therapy for the Egyptian population.

## METHODS

### Study Design

A static economic impact model was developed to compare LAIs of PP with daily oral antipsychotics (risperidone, olanzapine, and aripiprazole) for the treatment of patients with chronic schizophrenia. Our study included treatments used during relapse and hospitalization, validated by the local clinical practice, applied in the Health Insurance Organization (HIO) and Al-Azhar University hospitals and obtained from our expert panel. Individual responses were analyzed, gathered, and presented to reach a consensus through virtual meetings and phone interviews. This expert panel was composed of 3 psychiatrists affiliated with Al-Azhar University hospitals and HIO hospitals in Egypt. We collected insights from experts through a focus group by using the quasi-Delphi panel approach. The experts’ insights included the current local clinical practice and treatment patterns of these patients within the Egyptian representative healthcare settings (HIO and Al-Azhar University hospitals). We used a well-structured questionnaire to extract the insights from the panel. No ethics committee approval was required since this research did not include human subject data. Individual patient-level information was not used, and the research relied purely on secondary data. This model was developed using Microsoft Excel 365. Economic model validation, including both clinical and programming validation, was performed by using the International Society for Pharmacoeconomics and Outcomes Research (ISPOR) principles of good research practices for budget impact analysis.^16^

The PubMed and MEDLINE databases were comprehensively searched to identify English-language publications including clinical and economic data regarding the model structure, probabilities of the health states, and the treatment line options and doses. Articles that addressed the management of patients with chronic schizophrenia were selected on the basis of terms related to the clinical conditions and the budget impact of PP; these terms included “budget impact,” “schizophrenia,” “paliperidone palmitate,” “oral antipsychotics,” “randomized controlled trial,” “randomized,” “controlled trial,” “meta-analysis,” and “systematic review.” Twelve relevant articles and reports were identified by this electronic search and were reviewed; 5 articles were excluded as irrelevant.

Several assumptions were taken into consideration. It was assumed that the share of risperidone in the standard of care treatment was 35%, while for both olanzapine and aripiprazole, it was 32.5%. Furthermore, it was assumed that the risperidone standard of care is the use of generic oral products. It was also assumed that the same suicide prevalence was used in both arms but was linked with the nonadherence rate in each treatment arm, in accordance with the PRIDE study.^15^ All these assumptions were validated by the expert panel based on local clinical practice.

We conducted a budget impact analysis to compare the costs and consequences of LAI of PP with risperidone, olanzapine, and aripiprazole as one comparator arm (ie, standard of care in the HIO settings). The results of this analysis were expressed as a budget impact per patient per year and a cumulative budget impact over 2 years that included the total direct costs such as disease management (hospitalization), treatment-related costs (acquisition and monitoring), and indirect costs. The analysis was performed from the perspective of the HIO over a period of 2 years. A 2-year period was selected as the tender list was re-evaluated every 2 years in Egypt to inform and guide decision makers over a longer period. The total costs of both treatment arms were calculated by computing the unit costs calculated in Egyptian pounds (EGP) and multiplying it by the resource utilization (eg, drug dose, hospitalization rate, and follow-up visit frequency) according to the following equation:

Total Costs=Unit Costs(£)×Resource Utilization

### Target Population

**Figure 1.**
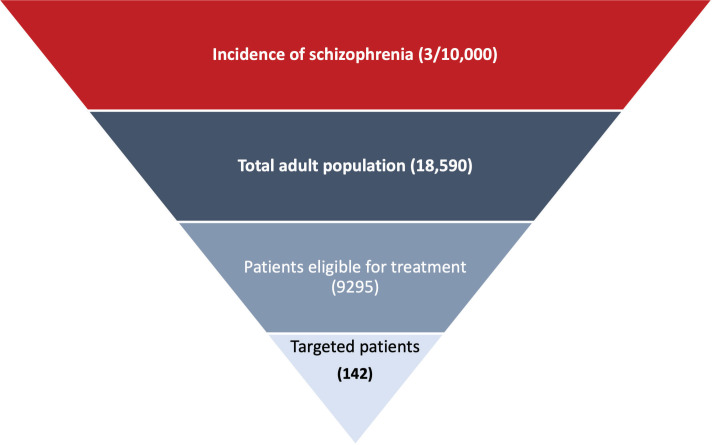
Target Population

**Figure 1** shows the target population of this study, which comprised adults 18 to 65 years old with chronic schizophrenia, a history of relapse, and a history of readmission/hospitalization as reported in the PRIDE study.^15^ Patients had been taken into custody by the criminal justice system at least twice in the previous 2 years, with at least 1 of these events leading to incarceration; released from most recent custody within 90 days of the screening visit; and accepted a once-monthly LAI antipsychotic.^15^ The patients did not use either clozapine within 3 months of screening or an injectable antipsychotic within 2 injection cycles of screening. Patients who had used intravenous drugs within 3 months of screening or had an opiate dependence disorder (DSM-IV) were excluded.^15^

The incidence of chronic schizophrenia was extracted from the literature and validated by the expert panel to reflect real-life clinical practice settings.^17^ The number of targeted patients was calculated by multiplying the number of the Egyptian adult population^18,19^ by the incidence of chronic schizophrenia in Egypt.^17^ The percentage of patients eligible to receive PP was estimated at 50%. The market penetration of PP was estimated at 2% based on the real-life practice validated by the expert panel. The target number of patients used in this study was estimated at 142 incident patients in both arms.

### Clinical Data

**Table 1** presents the model parameters. A static model (no transitions between any health state) was conducted; we measured all the costs and consequences associated with the 2 treatment arms, including hospitalization and follow-up. The clinical parameters for hospitalization and adherence to all treatments were extracted from the PRIDE trial,^15^ a prospective, open-label, randomized trial that took place in 50 medical centers in 25 US states and Puerto Rico. The primary end point was time to first treatment failure, defined as arrest/incarceration, psychiatric hospitalization, suicide, or increased psychiatric services to prevent hospitalization. The participants were adults aged 18 to 65 years with a current diagnosis of schizophrenia. The study compared LAI of PP to 7 oral antipsychotics (aripiprazole, haloperidol, olanzapine, paliperidone, perphenazine, quetiapine, and risperidone) over a 15-month treatment period. The annual medication-specific transition probabilities for hospitalization for all medications were validated based on a systematic review published by the National Institute for Health and Care Excellence (NICE) in the United Kingdom.^20^ The prevalence of suicide among patients who did not adhere to their medications was captured from our expert panel. All inputs for the selection, duration, and distribution of the subsequent treatments were also validated by the expert panel and represent the local clinical practice. The model did not include the most common side effects associated with the treatments due to their rare occurrence, and there was no need for resource utilization to manage them based on Egyptian local clinical practice.

**Table 1.** Model Inputs

**Parameter**	**Base Case**	**Low Value**	**High Value**	**Reference**
Clinical data				
Hospitalization with PP (%)	0.398	0.3582	0.4378	15
Hospitalization with oral antipsychotics (%)	0.54	0.4833	0.5907	15
Adherence with PP (%)	0.952	0.8568	1.0472	15
Adherence with oral antipsychotics (%)	0.243	0.2187	0.2673	15
Suicide with medication nonadherence (%)	0.15	0.135	0.165	Expert panel
Unit costs (in EGP)				
Hospitalization (per day)	370.00	296	444	UPA
Olanzapine, 5 mg	9.00	7.2	10.8	UPA
Aripiprazole, 10 mg	12.00	9.6	14.4	UPA
Electroconvulsive therapy	400.00	320	480	UPA
PP, 150 mg	1692	1430.288	2145.432	Manufacturer
PP, 100 mg	1787.860	0.208	0.312	Manufacturer
Oral risperidone, 3 mg	0.26	0.208	0.312	UPA
Renal function	80.00	64	96	UPA
Liver function	80.00	64	96	UPA
Prolactin level blood	100.00	80	120	UPA
CBC	40.00	32	48	UPA
Physician visits	150.00	120	180	UPA
Population data				
Incidence of schizophrenia	33/100 000	0.0002	0.0004	17
Egyptian population	102 000 000	81 600 000	122 400 000	18
% of Egyptian population 18-65 years old	60.75	48.6	72.9	19
Patients eligible to receive PP (%)	50	40	60	Expert panel
Patients receiving PP (%)	0.02%	0.01	0.02	Expert panel
Psychiatric work loss (days/mo)	6.0	4.80	7.20	3
Average daily wage (EGP)	216.7211591	173.38	260.07	22

Abbreviations: CBC, complete blood count; EGP, Egyptian pound; PP, paliperidone palmitate; UPA, Egyptian Authority for Unified Procurement, Medical Supply, and Management of Medical Technology.

### Costs

Direct medical costs and indirect costs were measured in our study over a 2-year period. Direct nonmedical costs were not considered because they are variable in Egypt, and there were no accurate data on their unit costs. We did not capture the intangible costs because no local data were available. The included direct medical costs are acquisition costs of medication, monitoring, and hospitalization costs. The unit cost of drug acquisition for all medications was extracted from the public sector, Egyptian Authority for Unified Procurement, Medical Supply and Management of Medical Technology (UPA) and multiplied by the utilization of each drug (dosing schedules extracted from PRIDE trial and validated from our local clinical practice). The dosage of PP was 150 mg intramuscular injection, as an initial injection, then 100 mg intramuscular injection, continued on a monthly maintenance dose. The length of stay in acute wards was assumed to be 90 days for both oral and LAI based on published articles and validated by local clinical practice.^21^ The cost for hospitalization, monitoring, and physician visits was also considered based on the average unit costs provided by the HIO and the university hospitals. The frequency of monitoring for each arm was provided by the expert panel. The monitoring tests included complete blood count, prolactin level blood, kidney function, and liver function tests. The costs due to subsequent treatments were considered in the relapse state and hospitalization. All unit costs were measured in 2022 EGP. The unit cost of each resource in **Table 1** was multiplied by its resource utilization to generate the total costs in each treatment arm (**Table 2**).

**Table 2.** Budget Impact Model Results

	**Costs (EGP)**		**Cost Difference (EGP)**
	**Without PP**	**With PP**	
Drug costs			
Year 1	724 004	2 764 329	2 040 326
Year 2	724 004	2 776 736	2 052 732
Total drug costs	1 448 008	5 541 066	4 093 058
Non-drug costs (medical and indirect costs)			
Year 1	5 632 865	3 063 056	-2 569 809
Year 2	5 632 865	3 063 056	-2 569 809
Total non-drug costs	11 265 730	6 126 111	- 5 139 619
Total drug and non-drug costs			
Year 1	6 356 869	5 827 385	- 529 484
Year 2	6 356 869	5 839 792	- 517 077
Total drug and non-drug costs	12 713 738	11 667 177	-1 046 561

Abbreviations: EGP, Egyptian pound; PP, paliperidone palmitate.

Drug costs constitute only a small fraction of the economic burden of schizophrenia, as most of the total costs are hospitalization and indirect costs due to productivity loss, unemployment, and early retirement. Indirect costs, including mortality and productivity loss, were measured. The productivity loss was measured by multiplying the Egyptian patient average wage per day by the psychiatric work loss days and the hospitalization rates for both treatment arms. The patient average hourly wage was captured by using the most recently published gross domestic product by the World Bank in 2020.^22^ Psychiatric work loss days were extracted from the US National Comorbidity Survey, conducted on 100 workers, to examine the association between recent *Diagnostic and Statistical Manual of Mental Disorders, Third Edition* (DSM-III-R) psychiatric disorders and work impairment in major occupational groups in the labor force.^3^ The mortality cost per year was measured by incorporating the prevalence rate of suicide for those who did not adhere to their medications in each treatment arm and multiplying it by the Egyptian patient average yearly wage.

### Statistical Analyses

Statistical analyses and all calculations were performed using Microsoft Excel 365. Two-sample comparisons were performed using 2-sided Student’s *t* tests for normally distributed variables to measure the statistical significance between the total costs of both treatment arms. A significance level of *P* < .05 was used.

### Sensitivity Analyses

One-way sensitivity analyses were performed to assure the robustness of the results. Various parameters were varied with 10% to 20% above or below their base case values. The parameters tested were the population, clinical, and cost data for each treatment arm. Two-way sensitivity analyses were not conducted because there is no clear correlation between the parameters tested.

## RESULTS

The target population in our model was estimated to be 142 patients. In the first year, the total drug costs when LAI of PP was compared with oral antipsychotics were £2.7 million and £724 004 (incremental cost, £2 040 326), respectively, while the total medical and indirect costs of LAI of PP and oral antipsychotics were £3 million and £5.6 million (cost savings, £2 569 809), respectively (**Table 2**). In the second year, the total drug costs for LAI of PP and oral antipsychotics were £2.7 million and £724 004 (incremental cost, £2 052 732), respectively (**Figure S1**), while the total medical and indirect costs of LAI of PP and oral antipsychotics were £3 million and £5.6 million (cost savings, £2 569 809), respectively (**Figure S2**).

**Figure 2.**
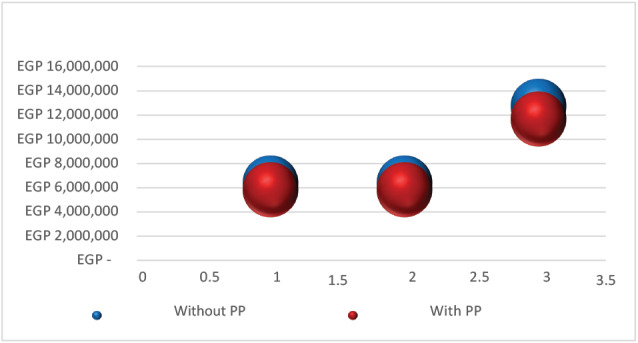
Total Direct and Indirect Costs With and Without Paliperidone Palmitate Abbreviations: EGP, Egyptian pound; PP, paliperidone palmitate.

The total costs of LAI of PP (£11.6 million) over 2 years were less than those of oral antipsychotics without PP (£12.7 million). LAI of PP produced an estimated budget savings of £1 046 561 (budget savings per patient per year, £3667), as shown in **Figure 2**. A statistically significant difference was shown in the total costs in years 1 and 2 between both treatment arms (*P* = .0001). **Table 3** showed all the cost components (treatment costs, disease management costs, and indirect costs) in both treatment arms.

**Table 3.** Total Cost Components in Both Treatment Arms

	**Total Costs (EGP)**		**Cost Difference (EGP)**
	**Without PP**	**With PP**	
Treatment costs	1 448 008	5 541 066	4 093 058
Disease management costs (medical)	5 575 061	3 659 965	(1 915 096)
Indirect costs	5 690 669	2 466 146	(3 224 523)

Abbreviations: EGP, Egyptian pound; PP, paliperidone palmitate.

On the other hand, LAIs of PP resulted in 18 hospitalizations avoided per year in comparison to the without-PP arm. Thus, it is estimated that 36 total hospitalizations were avoided with PP over 2 years and hence less burden on hospitals and healthcare staff.

### Sensitivity Analysis

**Figure 3.**
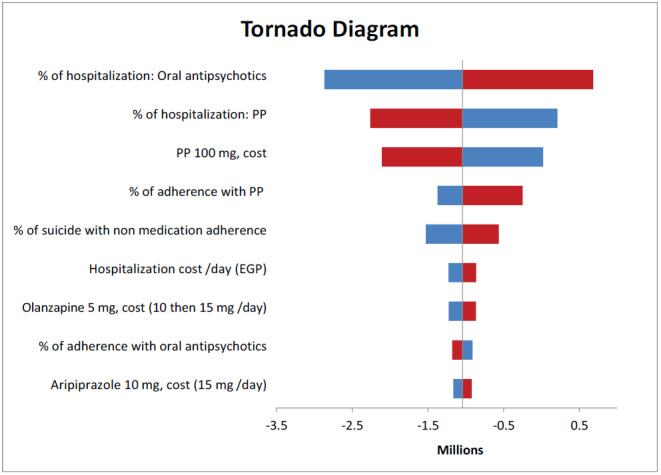
One-way Sensitivity Analysis of Paliperidone Palmitate vs Without Paliperidone Palmitate Abbreviation: PP; paliperidone palmitate.

Deterministic sensitivity analyses were conducted to explore the effect of changes in model parameters on the direct medical and indirect costs over the time horizon. The tornado diagram showed the most sensitive parameters (**Figure 3**). The diagram revealed that the percentage of hospitalizations for both oral antipsychotics and PP had the greatest impact on the total costs. Thus, real-world data need to be collected from local hospitals to ensure the robustness of the results.

## DISCUSSION

Stigma and discrimination of human rights of patients with schizophrenia is common worldwide.^23^ To promote equity of access to mental health services, the Egyptian government is encouraging the development of community-based psychiatric units and outpatient facilities in Egypt.^2^ Thus, people with schizophrenia can manage their disorder effectively, and valuable treatments can be adopted that can maximize patients’ outcomes and decrease the financial burden of the disease and its complications. This is especially important because schizophrenia is considered the most expensive psychiatric disorder worldwide.^24^

Our study shows the savings generated from the use of LAI of PP compared with oral risperidone in the treatment of chronic schizophrenia in the HIO and Al-Azhar University hospitals. PP shows the social and clinical benefit of LAI in terms of reduction of aggressive behavior or better community functioning,^15^ which also potentially resulted in cost savings and better productivity. Debaveye et al conducted an economic evaluation to model the health impacts of the pharmaceutical supply chain, administration and disposal of the drug, and patient benefit within a 1-year period in terms of DALYs.^25^ They modeled 3 patient groups: medicine coverage of PP for either 1 month (PP1M) or 3 months (PP3M) at a time, and compared them with treatment interruption as a control group. They concluded that the overall burden was lower for PP1M and PP3M treatment than treatment interruption because patients are kept more stable and less hospitalized (the human health burden was outweighed by the human health benefit).^25^

The consequences of nonadherence include increased risk of hospitalization, incomplete remission, impaired education and occupational performance, lower quality of life, suicidality and self-harm behavior, aggressivity, substance misuse, and increased costs of treatment. In addition, this disease begins in young adulthood, which can affect the patient over their lifetime, negatively impacting their working ability and limiting their opportunities in the labor market. Thus, the benefits of PP are not restricted solely to overcoming the problem of nonadherence; they also allow the clinician to identify true lack of response (often difficult to evaluate in the case of partial or total nonadherence to oral medications) and may foster more regular contact with caregivers. Moreover, they have better bioavailability, avoid first-pass metabolism, establish more stable concentrations and a more predictable correlation between dosages and plasma levels, and reduce the risk of voluntary overdose.^26^

Our study results were consistent with a budget impact model conducted in Austria to estimate the budget effects of the introduction of PP in schizophrenia. This study concluded that the introduction of PP in Austria was budget neutral.^27^ Another budget impact analysis of change in reimbursement policy using a prevalence-based model over a 5-year time horizon, conducted in Japan, revealed that an additional reimbursement for the use of PP in schizophrenia patients was likely to be cost-neutral/cost-saving compared with olanzapine, risperidone and aripiprazole and should be considered as a policy option to improve patient outcomes and budget sustainability.^28^ A further budget impact study conducted in Indonesia estimated the percentage of patients seeking care, treatment patterns, and quantities of medications dispensed for schizophrenia patients in 3 kinds of health facilities: mental health hospital, general hospital, and health center.^29^ The study concluded that the addition of PP may increase the total cost, but it could be an option for schizophrenia that could lower the total number of relapses.^29^

This study has several notable strengths. First, we extracted the clinical parameters from real-world, randomized multicenter studies that used strong evidence and were free of bias. Second, it is the first budget impact model conducted in Egypt measuring all cost consequences of PP compared with the standard of care in chronic schizophrenia in Egyptian representative healthcare settings. Third, we measured the direct and indirect costs to demonstrate the real value of the medications in schizophrenia and how their patient and economic impact from a societal perspective.

Our study also had some limitations. First, cost data of informal care for people with schizophrenia were not measured because they are not available in Egypt. Second, we have not evaluated the utilization of outpatient health services, although there might be some additional cost savings in this sector as well. Third, our results might be underestimated because the reduced risk of self-harm, a factor that improves long-term clinical outcomes, was not measured.^30^ Fourth, our economic analysis omitted common side effects of antipsychotic treatment that may cause impairments in quality of life (eg, sexual dysfunction, increase in prolactin levels, and cardiovascular and gastrointestinal side effects).

Certain key drivers of the total costs were identified, and sensitivity analysis was performed to estimate the overall impact on costs and savings. Therefore, the major drivers of the budget impact are the percentage of hospitalizations for both arms due to relapse, PP price, and percentage of patient adherence to PP. The use of PP to treat chronic schizophrenia provides a clear picture of how it can influence patient outcomes and reduce the burden on medical communities and hospitals.

## CONCLUSION

The lower hospitalization rates with PP offset the increase in drug costs. PP may potentially be cost-saving compared with the standard of care in chronic schizophrenia in the Egyptian representative healthcare settings. This result is robust to variations in all parameters. Policy makers should consider this approach to improve patient outcomes and budget sustainability.

## Supplementary Material

Supplementary Online Material

## References

[ref-235761] Rehm Jürgen, Shield Kevin D. (2019). Global burden of disease and the impact of mental and addictive disorders. Current Psychiatry Reports.

[ref-235762] Ghanem M., Gadallah M., Meky F.A., Mourad S., El Kholy G. (2009). National Survey of Prevalence of Mental Disorders in Egypt: preliminary survey. Eastern Mediterranean Health Journal.

[ref-235763] Kessler RONALD C., Frank RICHARD G. (1997). The impact of psychiatric disorders on work loss days. Psychological Medicine.

[ref-235764] Nasrallah H.A., Goldberg J.F., Correll C.U., SAD Working Group (2010). Differential diagnosis and therapeutic management of schizoaffective disorder. Ann Clin Psychiatry.

[ref-235765] Baillargeon Jacques, Binswanger Ingrid A., Penn Joseph V. , Williams Brie A., Murray Owen J. (2009). Psychiatric disorders and repeat incarcerations: the revolving prison door. American Journal of Psychiatry.

[ref-235766] Jääskeläinen E., Juola P., Hirvonen N., McGrath J. J., Saha S., Isohanni M., Veijola J., Miettunen J. (2013). A systematic review and meta-analysis of recovery in schizophrenia. Schizophrenia Bulletin.

[ref-235767] Leucht Stefan, Tardy Magdolna, Komossa Katja, Heres Stephan, Kissling Werner, Salanti Georgia, Davis John M (2012). Antipsychotic drugs versus placebo for relapse prevention in schizophrenia: a systematic review and meta-analysis. The Lancet.

[ref-235768] Laursen Thomas Munk, Nordentoft Merete, Mortensen Preben Bo (2014). Excess early mortality in schizophrenia. Annual Review of Clinical Psychology.

[ref-235769] Karagianis J., Novick D., Pecenak J., Haro J. M., Dossenbach M., Treuer T., Montgomery W., Walton R., Lowry A. J. (2009). Worldwide-Schizophrenia Outpatient Health Outcomes (W-SOHO): baseline characteristics of pan-regional observational data from more than 17,000 patients. International Journal of Clinical Practice.

[ref-235770] National Collaborating Centre for Mental Health. Schizophrenia (2010). The NICE Guideline on Core Interventions in the Treatment and Management of Schizophrenia in Adults in Primary and Secondary Care.

[ref-235771] Haddad Peter M., Brain Cecilia, Scott Jan (2014). Nonadherence with antipsychotic medication in schizophrenia: challenges and management strategies. Patient Related Outcome Measures.

[ref-235772] Ascher-Svanum Haya, Peng Xiaomei, Faries Douglas, Montgomery William, Haddad Peter M (2009). Treatment patterns and clinical characteristics prior to initiating depot typical antipsychotics for nonadherent schizophrenia patients. BMC Psychiatry.

[ref-235773] Leucht Claudia, Heres Stephan, Kane John M., Kissling Werner, Davis John M., Leucht Stefan (2011). Oral versus depot antipsychotic drugs for schizophrenia—a critical systematic review and meta-analysis of randomised long-term trials. Schizophrenia Research.

[ref-235774] Kishimoto T., Robenzadeh A., Leucht C., Leucht S., Watanabe K., Mimura M., Borenstein M., Kane J. M., Correll C. U. (2014). Long-acting injectable vs oral antipsychotics for relapse prevention in schizophrenia: a meta-analysis of randomized trials. Schizophrenia Bulletin.

[ref-235775] Alphs Larry, Benson Carmela, Cheshire-Kinney Kimberly, Lindenmayer Jean-Pierre, Mao Lian, Rodriguez Stephen C., Starr H. Lynn (2015). Real-world outcomes of paliperidone palmitate compared to daily oral antipsychotic therapy in schizophrenia: a randomized, open-label, review board-blinded 15-month study. The Journal of Clinical Psychiatry.

[ref-235776] Mauskopf Josephine A., Sullivan Sean D., Annemans Lieven, Caro Jaime, Mullins C. Daniel, Nuijten Mark, Orlewska Ewa, Watkins John, Trueman Paul (2007). Principles of good practice for budget impact analysis: report of the ISPOR task force on good research practices—budget impact analysis. Value in Health.

[ref-235777] Fouad Amira A, Fawzi Mohab M, El Masry N. (2013). Psychosocial burden among caregivers of patients with schizophrenia in Egypt. Zagazig University Medical Journal.

[ref-235778] World Bank, Country Profile, Egypt: World Development Indicators database.

[ref-235779] Statista Egypt: Age structure from 2010 to 2020.

[ref-235780] National Collaborating Centre for Mental Health (2014). Schizophrenia. The psychosis and schizophrenia in adults. The NICE Guideline on Treatment and Management.

[ref-235781] Kasahara-Kiritani Mami, Chaturvedi Amish, Inagaki Ataru, Wakamatsu Akihide, Jung Wonjoo (2020). Budget impact analysis of long acting injection for schizophrenia in Japan. Journal of Medical Economics.

[ref-235782] World Bank national accounts data, and OECD National Accounts data files. GDP per capita (current LCU) - Egypt, Arab Rep.

[ref-235783] World Health Organization, Schizophrenia.

[ref-235784] Rossler Wulf, Salize Hans Joachim, van Os Jim, Riecher-Rössler Anita (2005). Size of burden of schizophrenia and psychotic disorders. European Neuropsychopharmacology.

[ref-235785] Debaveye Sam, De Smedt Delphine, Heirman Bert, Kavanagh Shane, Dewulf Jo (2019). Human health benefit and burden of the schizophrenia health care pathway in Belgium: paliperidone palmitate long-acting injections. BMC Health Services Research.

[ref-235786] Olivares José M, Pinal Beatriz, Cinos Carmen (2011). Comparison of long-acting antipsychotic injection and oral antipsychotics in schizophrenia. Neuropsychiatry.

[ref-235787] Ransmayr Sara, Mehnert Angelika, Mahlich Jörg (2013). Budget Impact Analyse von Paliperidon Palmitat im österreichischen Versorgungskontext. PharmacoEconomics German Research Articles.

[ref-235788] Kasahara-Kiritani Mami, Chaturvedi Amish, Inagaki Ataru, Wakamatsu Akihide, Jung Wonjoo (2020). Budget impact analysis of long acting injection for schizophrenia in Japan. Journal of Medical Economics.

[ref-235789] Puspandari D.A., Hafidz F., Tsalatsita R.M. (2019). Budget impact analysis of schizophrenia treatment in Indonesia. Value in Health.

[ref-235790] Lambert M., De Marinis T., Pfeil J., Naber D., Schreiner A. (2010). Establishing remission and good clinical functioning in schizophrenia: predictors of best outcome with long-term risperidone long-acting injectable treatment. European Psychiatry.

