# Supplementary material for: Projecting the Potential Budget Impact Analysis of Paliperidone Palmitate in Egyptian Adult Patients with Schizophrenia
Source: J Health Econ Outcomes Res. 2023 Aug 17;10(2):23–9. doi: 10.36469/001c.83240 (PMC10439680; doi:10.36469/001c.83240)
Supplement: Supplementary Online Material [file jheor_2023_10_2_83240_177119.pdf]

Journal of Health Economics and Outcomes Research

Online Supplementary Material

Projecting the Potential Budget Impact Analysis of Paliperidone Palmitate in Egyptian Adult Patients With Schizophrenia. *JHEOR*. 2023;10(2):23-29. doi:10.36469/jheor.2023.83240

Figure S1: Drug Costs of Paliperidone Palmitate vs Without Paliperidone Palmitate

Figure S2: Non-drug Costs of Paliperidone Palmitate vs Without Paliperidone Palmitate

This supplementary material has been provided by the authors to give readers additional information about their work.

This is an open-access article distributed under the terms of the Creative Commons Attribution 4.0 International License (CCBY-4.0). View this license's legal deed at http://creativecommons.org/licenses/by/4.0 and legal code at http://creativecommons.org/licenses/by/4.0/legalcode for more information.

Figure S1. Drug Costs of Paliperidone Palmitate vs Without Paliperidone Palmitate

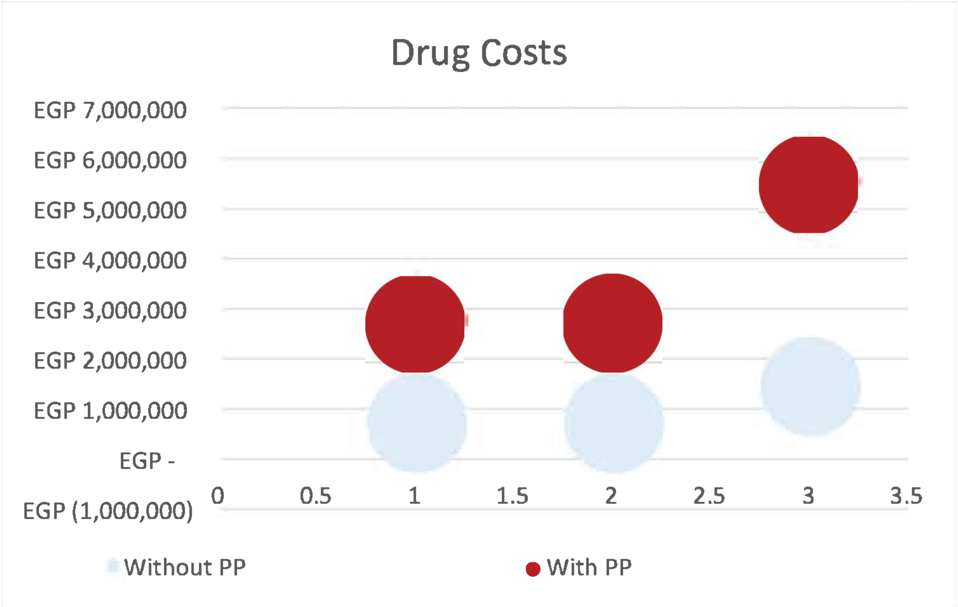

Abbreviations: EGP, Egyptian pound; PP, paliperidone palmitate.

Figure S2. Non-drug Costs of Paliperidone Palmitate vs Without Paliperidone Palmitate

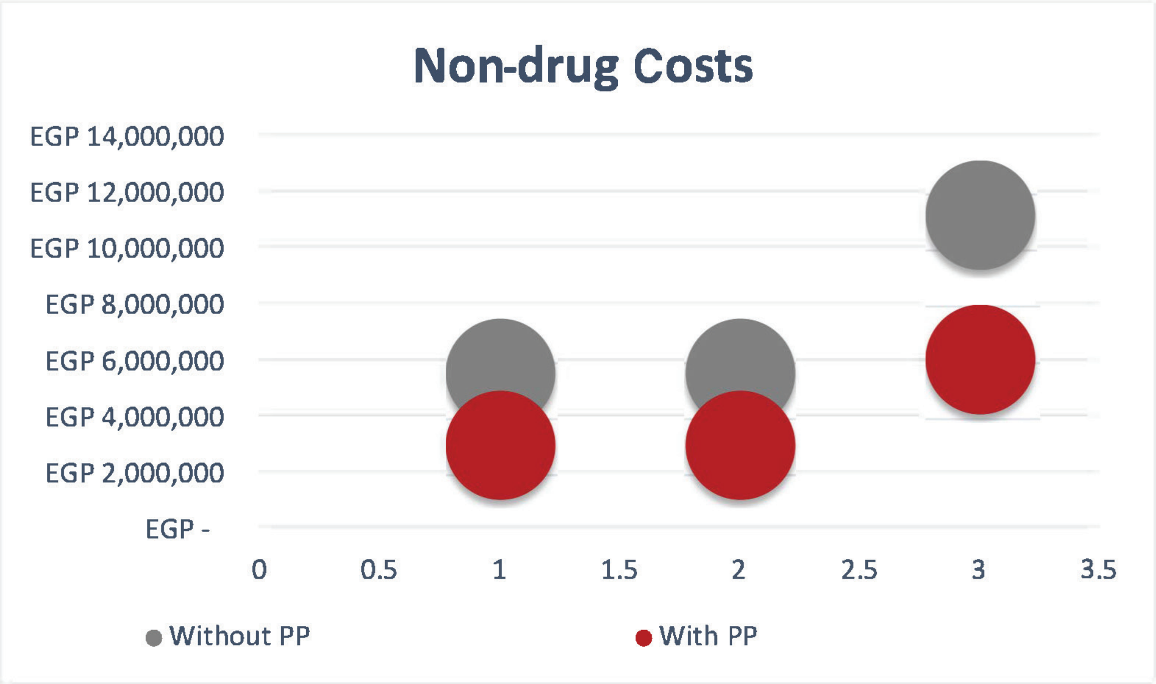

Abbreviations: EGP, Egyptian pound; PP, paliperidone palmitate.
